# Assessing Earthquake Forecast Performance Based on *b* Value in Yunnan Province, China

**DOI:** 10.3390/e23060730

**Published:** 2021-06-08

**Authors:** Rui Wang, Ying Chang, Miao Miao, Zhiyi Zeng, Hongyan Chen, Haixia Shi, Danning Li, Lifang Liu, Youjin Su, Peng Han

**Affiliations:** 1 Department of Earth and Space Science, Southern University of Science and Technology, Shenzhen 518055, China; 11930870@mail.sustech.edu.cn (R.W.); miaom@sustech.edu.cn (M.M.); 11930855@mail.sustech.edu.cn (Z.Z.); 11930858@mail.sustech.edu.cn (H.C.); 2 Institute of Mining Engineering, BGRIMM Technology Group, Beijing 100160, China; changy@bgrimm.com; 3 China Earthquake Networks Center, Beijing 100045, China; shihaixia08@seis.ac.cn; 4 Earthquake Administration of Yunnan Province, Kunming 650224, China; zuni_2001@163.com (D.L.); lifang_l@sina.com (L.L.); suyoujin0818@sina.com (Y.S.)

**Keywords:** *b* value, molchan error diagram, earthquake forecast, Yunnan, China

## Abstract

Many studies have shown that *b* values tend to decrease prior to large earthquakes. To evaluate the forecast information in *b* value variations, we conduct a systematic assessment in Yunnan Province, China, where the seismicity is intense and moderate–large earthquakes occur frequently. The catalog in the past two decades is divided into four time periods (January 2000–December 2004, January 2005–December 2009, January 2010–December 2014, and January 2015–December 2019). The spatial *b* values are calculated for each 5-year span and then are used to forecast moderate-large earthquakes (M ≥ 5.0) in the subsequent period. As the fault systems in Yunnan Province are complex, to avoid possible biases in *b* value computation caused by different faulting regimes when using the grid search, the hierarchical space–time point-process models (HIST-PPM) proposed by Ogata are utilized to estimate spatial b values in this study. The forecast performance is tested by Molchan error diagram (MED) and the efficiency is quantified by probability gain (*PG)* and probability difference (*PD)*. It is found that moderate–large earthquakes are more likely to occur in low *b* regions. The MED analysis shows that there is considerable precursory information in spatial *b* values and the forecast efficiency increases with magnitude in the Yunnan Province. These results suggest that the *b* value might be useful in middle- and long-term earthquake forecasts in the study area.

## 1. Introduction

The Gutenberg–Richter (G–R) law describes the magnitude–frequency relationship of earthquakes, which is lgN=a−bM [1,2]. In the formula, constant *a* reflects the seismicity of the region, and constant *b* indicates the relative ration of small to large earthquakes. The *b* value has been proved to be inversely related to the underground stress by laboratory experiments and seismic studies [3,4,5,6,7]. Low *b* values are likely to correspond to high stress states and a decrease in the *b* value may indicate an increase in stress. Hence the *b* value could be an indicator of the stress level underground and may have potential value in earthquake risk assessments. Due to the self-similarity of the earthquake source, recently, it has been documented in several publications of natural time analysis of earthquake catalogues that the entropy concept is of key importance in order to achieve an earthquake risk assessment [8,9,10] since it was revealed that a decrease in the *b*-value before large earthquakes reflects an increase in the order parameter fluctuations upon approaching the critical point (mainshock) stemming from both origins of self-similarity [11], i.e., the process increments infinite variance and/or process memory [12]. Until now, the *b* value has been widely applied in natural seismic research and recently extended to activity analysis of induced earthquakes [7,13,14,15,16,17,18,19,20,21,22,23,24,25,26,27,28,29,30,31,32,33,34,35,36,37]. It was reported that large earthquakes tend to occur in areas with low *b* values [25,26], and the temporal variations of *b* values in epicenter regions show a decrease trend before major earthquakes [24,25,26,32], such as the 2011 Mw9.0 Tohoku earthquake, the 2004 Mw9.0 Sumatra earthquake, and the 2008 Mw7.9 Wenchuan earthquake [36,38,39]. Although precursory *b* value changes have been claimed in many case studies, systematic assessment of forecast performance based on *b* value is still rare. To verify the feasibility of *b* values for earthquake forecasting and regional risk assessment, we apply statistical investigation, using the Molchan error diagram (MED) in Yunnan Province of China, where the fault system is complex and the seismicity is intense. 

The conventional method to calculate the spatial *b* value is the grid search, using the set of seismic events closest to the grid points with a fixed number, a fixed radius [14,24,25,31,32,40,41,42,43,44], or an adaptive window, changing its size and shape to take into account the differences in the statistical estimates of *b* values in adjacent grid nodes [45,46]. As a statistical method, to ensure the reliability of results, the calculation of the *b* value requires complete samples with appropriate numbers [26,47], and it is suggested that there should be at least two orders of both earthquake numbers and magnitudes to obtain a robust *b* value estimation [48]. In practice, to obtain higher spatial resolution, there would be an overlap of the seismic events used to calculate the *b* value of adjacent grid points [47,49]. On the other hand, the *b* value in different faulting regimes may vary a lot [16,17,25], which can cause large biases when earthquakes on different faults are allotted in the same dataset for b value computation. 

Ogata proposed the hierarchical space–time point-process models (HIST-PPM), cubic B-spline expansions and the Bayesian method for estimation and interpolation of *b* values in space [33,34,35,50,51,52,53]. As it does not require allocation of an earthquake sample, it may have advantages in computing spatial *b* values, particularly in highly fractured regions with complex fault systems, such as Yunnan Province, China. Therefore, in this study, we apply the HIST-PPM method to earthquake catalogs during 2000–2020 in Yunnan to evaluate whether the *b* value is useful in mid- and long-term earthquake forecasts in the region.

## 2. Data and Methods

### 2.1. Data

Due to the relative motion of the Indian Ocean plate to the Eurasian plate, earthquakes occur frequently in the Yunnan region. In history, several devastating earthquakes have occurred, and the seismic risk is currently very high [54,55]. It is important to conduct mid- and long-term earthquake forecasts in this area for disaster mitigation and relief supplies preparation. 

**Figure 1:**
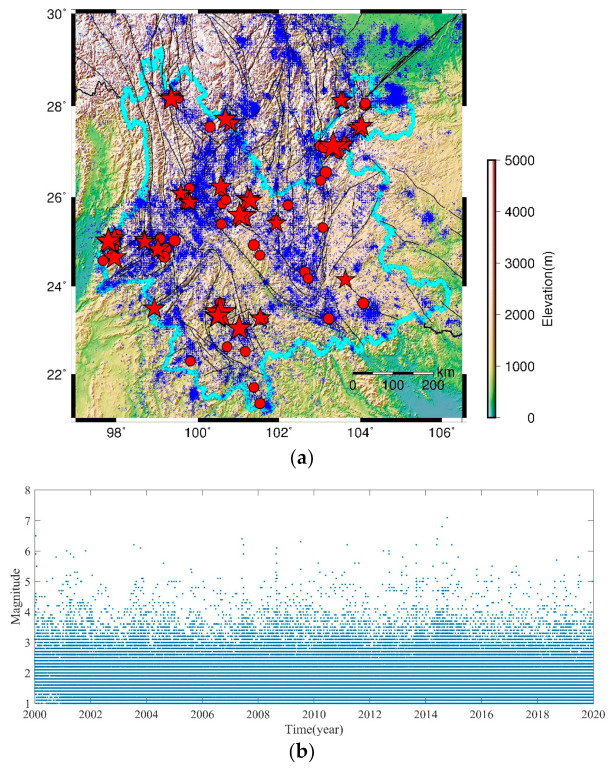
The spatial and temporal distribution of earthquakes in Yunnan from 2000 to 2019. (**a**) Map of Yunnan and earthquake distributions. The light blue line is the provincial boundary, and the dark blue circles are earthquakes. The red dots present the events in Yunnan province with 5.0≤M<5.5 and the red stars show M≥5.5 events. The size of the symbol is scaled to magnitude. The black lines indicate main faults [57]. (**b**) Temporal distribution of the earthquakes shown in Figure 1a.

The catalog in this study is provided by Earthquake Administration of Yunnan Province China. The research area covers 97° E–106.5° E and 21° N–30° N, including the whole Yunnan Province bound by a light blue line, as shown in Figure 1a. We chose the catalog from the year 2000 because the seismic network was upgraded then [56]. The spatial distributions of the earthquakes during 2000–2019 are shown in Figure 1a, and the temporal variation is given in Figure 1b. 

Early studies suggested that the *b* value dropped to a relatively lower level about 3–5 years before the mainshock [36,38]. As our attempt, we divide the catalog during 2000–2019 into four time periods (January 2000–December 2004, January 2005–December 2009, January 2010–December 2014, and January 2015–December 2019). Each period includes 5 years. To evaluate the magnitude of completeness (*Mc*) in space, we divide the study region into 0.1° × 0.1° grids. Several approaches for *Mc* estimation have been proposed, such as the entire magnitude range (EMR) method [50,58], maximum curvature (MAXC) method [59,60,61], goodness-of-fit test (GFT) method [59], *Mc* by *b*-value stability (MBS) method [62], and median based analysis of the segment slope (MBASS) method [63]. Woessner and Wiemer (2005) compared the EMR method with GFT, MBS and MAXC, finding that EMR showed a superior performance when applied to synthetic test cases or real data from regional and global earthquake catalogues [58]. The EMR method, however, is also the most computationally intensive.

In this study, we use the MAXC method in combination with the bootstrap. Earthquake events within a distance *r* from the grid center is selected first and then a bootstrap is applied to the selected sample. We repeat 1000 times the bootstrap and obtain 1000 *Mcs* for each grid. The *Mc* of the grid is determined by the mean value. In practice, a small *r* can reduce overlap events between adjacent grid points and increase spatial resolution. However, there might be not enough earthquake samples when r is too small, which may reduce the stability of *Mc*. To make a tradeoff between stability and spatial resolution, we set r = 50 km. 

**Figure 2:**
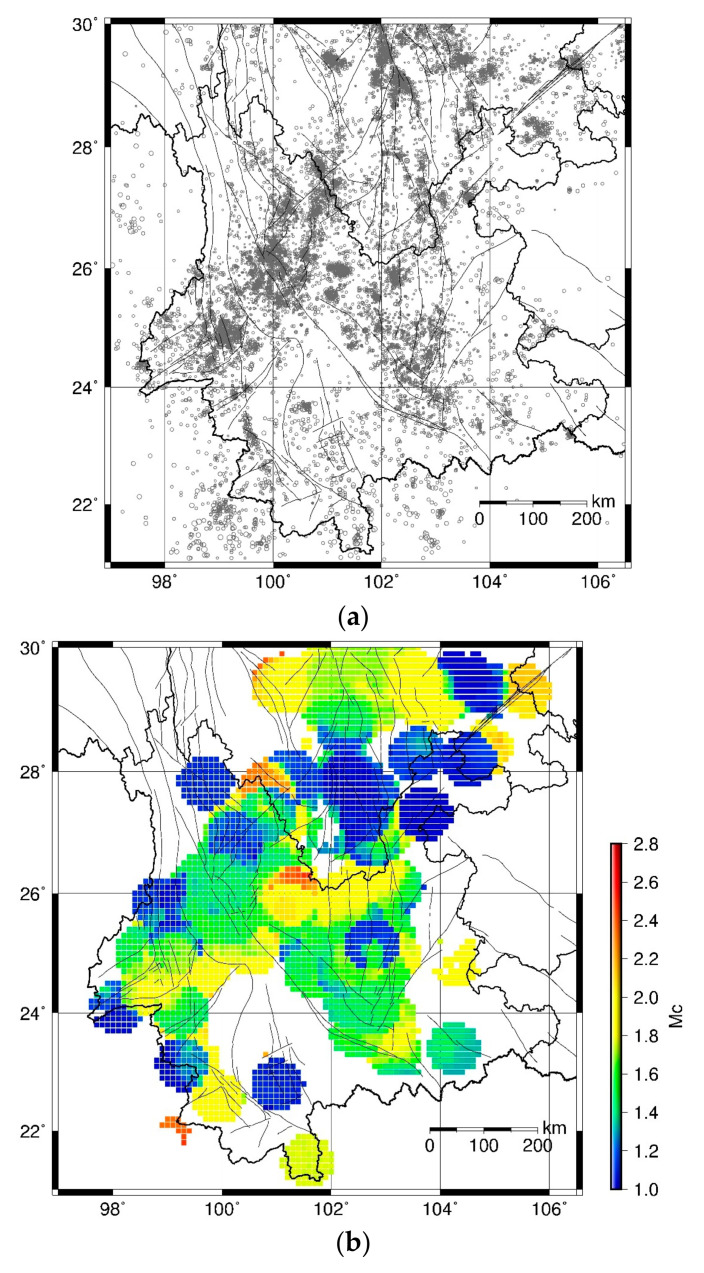

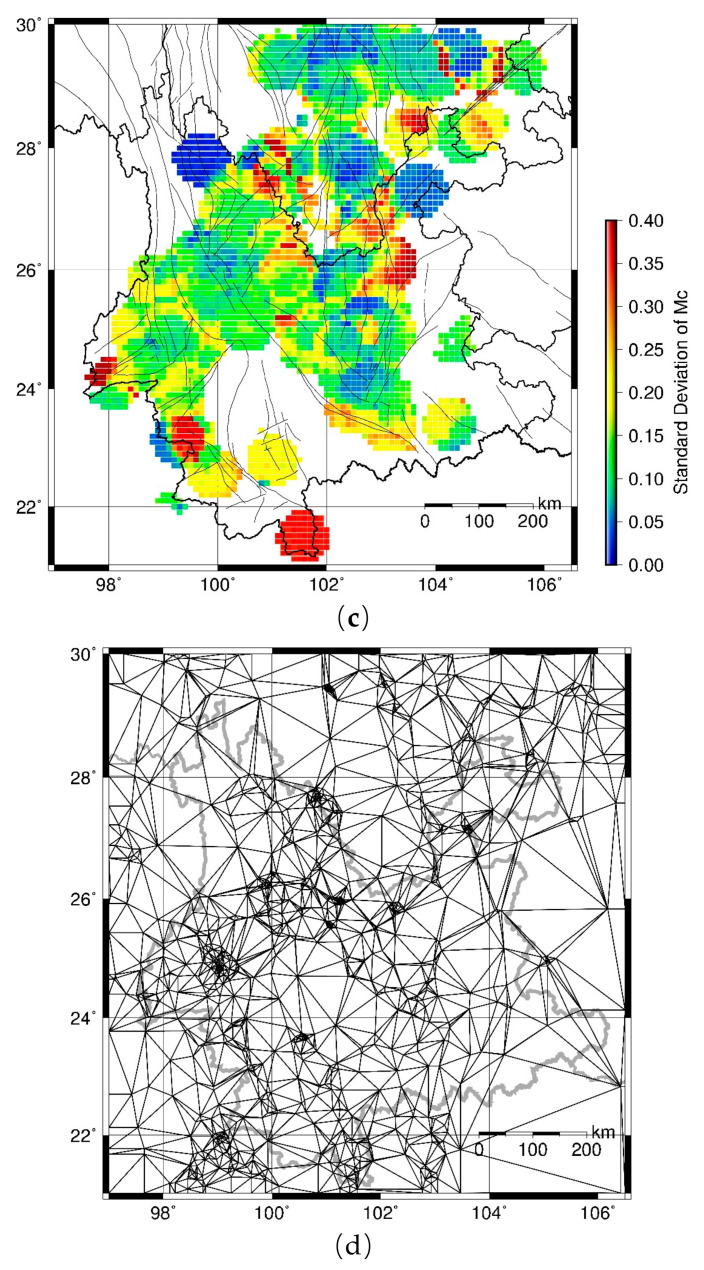
(**a**) Earthquake distribution during January 2000–December 2004. The size of the circle is scaled to the magnitude; (**b**) spatial distribution of Mc during January 2000–December 2004; (**c**) spatial distribution of standard deviation of *Mc* during January 2000–December 2004; (**d**) Delaunay triangle tessellation connecting the epicenters of M≥3.0 events during January 2000–December 2004.

Taking the catalog from January 2000 to December 2004 as an example, the seismicity is shown in Figure 2a and the *Mc* distribution is shown in Figure 2b. If the number of events for a grid is less than 100, the *Mc* of the grid is set as not available. The grids with available *Mc* results cover most of Yunnan province, with maximum *Mc* = 2.6 and maximum standard deviation = 0.4 (Figure 2c). As the seismic network keeps upgrading and the detectability improves with time, the *Mc* has decreased since 2000 [64]. Therefore, we set *Mc* = 3.0 and it can ensure that the earthquake with a magnitude above *Mc* is complete throughout the entire analyzed period in Yunnan Province. Next, we use the earthquakes above *Mc* to compute the *b* value. 

### 2.2. b Value Estimation

Gutenberg and Richter revealed the magnitude–frequency relationship (G–R law) as follows:

*lgN* = *a* − *bM (M ≥ Mc)* (1)

where *N* is the number of events with M≥Mc, *a* and *b* are constant.

Based on the G–R law, the number of earthquakes is defined in terms of the conditional intensity function as follows:

(2) $$N\left(M\right)=10^{a-b\left(M-M_{c}\right)}=Ae^{-\beta \left(M-M_{c}\right)}$$

where β=bln10. The probability density distribution of magnitude can be derived as the following:

(3) $$f\left(M\right)=\frac{N\left(M\right)}{\int_{M_{c}}^{\infty }N\left(M\right)dM}=\beta e^{-\beta \left(M-M_{c}\right)}$$

The likelihood function to a set of earthquakes events with independent magnitudes ($M_{1},M_{2},\ldots \ldots ,M_{n}$) is as follows [40]:

(4) $$L\left(\beta \right)=\overset{n}{\underset{i=1}{\prod }}f_{\beta }\left(M_{i}\right)=\overset{n}{\underset{i=1}{\prod }}\beta e^{-\beta \left(M_{i}-M_{c}\right)}$$

Ogata considered that the β value depended on location or/and time and proposed the hierarchical space–time point-process models (HIST-PPM) [34,35,65]. In this study, it is assumed that β is a function of epicenter $\left(x_{i},y_{i}\right)$ in a way such way that the following is true:

(5) $$\beta =\beta \left(x_{i},y_{i}\right)$$

Since the *b* value is positive, the parametrization of the function $\beta \left(x_{i},y_{i}\right)$ is carried out by the following:

(6) $$\beta \left(x,y\right)=e^{\phi_{\theta }\left(x,y\right)}$$

where the $\phi_{\theta }$ is the 2D B-spline function, and θ is the coefficient of function $\phi_{\theta }$ [35]. In this way, β is represented by a flexible function of location [34,35,50,51,52,66]. 

In HIST-PPM, Ogata tessellated the study space by the Delaunay triangle apexing at epicenters of seismic events, then estimated the parameter θ by maximizing the penalized log-likelihood as follows [35,65]:

(7) $$R\left(\theta |w\right)=lnL\left(\theta \right)-Q\left(\theta |w\right)$$

The Q(θ|w) is the penalty term, defined as the following:

(8) $$Q\left(\theta |w\right)=w\iint \left\{{\left(\frac{\partial \phi_{\theta }\left(x,y\right)}{\partial x}\right)}^{2}+{\left(\frac{\partial \phi_{\theta }\left(x,y\right)}{\partial y}\right)}^{2}\right\}dxdy$$

where w is the weight to be optimized by Akaike’s Bayesian Information Criterion (*ABIC*) [35,67]. Based on the entropy maximization principle [67,68], Akaike (1980) developed Good’s method and defined the *ABIC* as follows [69]:

(9) $$ABIC=-2max\left(\log L\right)+2\left(number\;of\;\mathrm{hyperparameters}\right)$$

The hyperparameters with a smaller *ABIC* value provides a better fit to data [35]. More details about the model fitting are given in the manual of HIST-PPM [65].

After obtaining *b* values on the mesh points, the values in each triangle can be computed by linear interpolation of the *b* values at the triangle vertices. By this method, we get the spatial *b* value with $0.1^{{}^{\circ}}\times 0.1^{{}^{\circ}}$ resolution in this study.

## 3. Results

### 3.1. Spatial b Value and Forecast Performance in Each 5-Year Time Period

**Figure 3:**
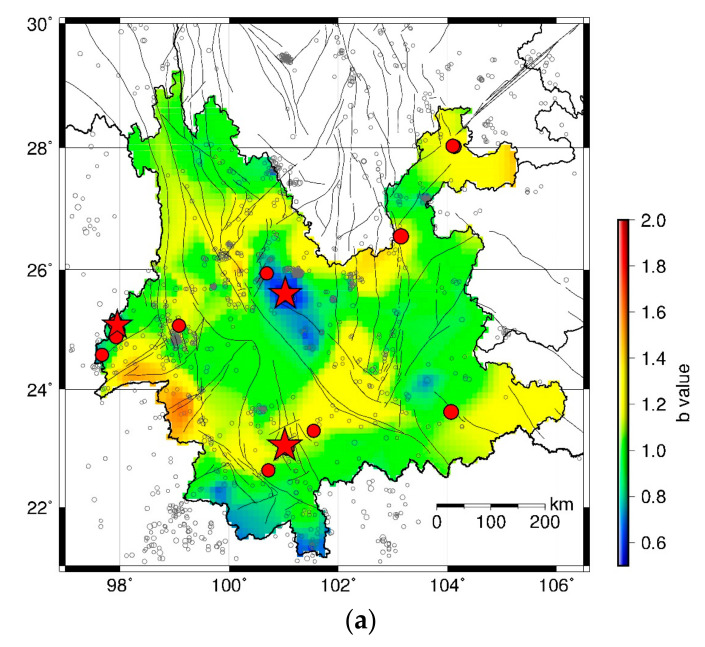

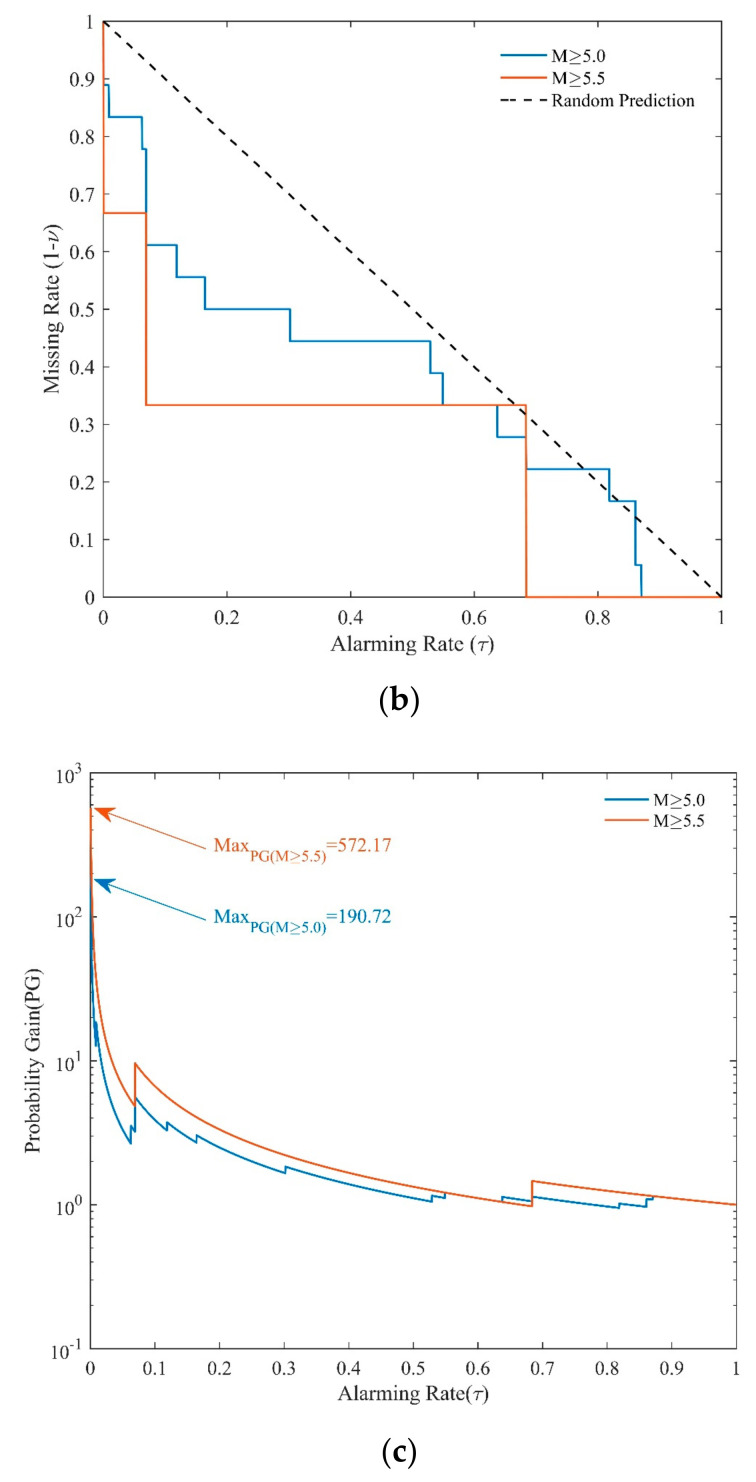
(**a**) Spatial distribution of *b* values in January 2000–December 2004. Black circles present the earthquake events with M ≥ Mc during January 2000–December 2004. Red dots show locations of events with 5.0≤M<5.5 and red stars show locations of events with M≥5.5 in Yunnan province during January 2005–December 2009. The size of symbol is scaled to the magnitude. (**b**) MED of forecast performance using the *b* values in (**a**). The earthquake number $N_{M\geq 5.0}=18$ and $N_{M\geq 5.5}=3$. The blue line gives the result for M≥5.0 events and the red line shows the result for M≥5.5. (**c**) The PG variations of the predictions in (**b**).

**Figure 4:**
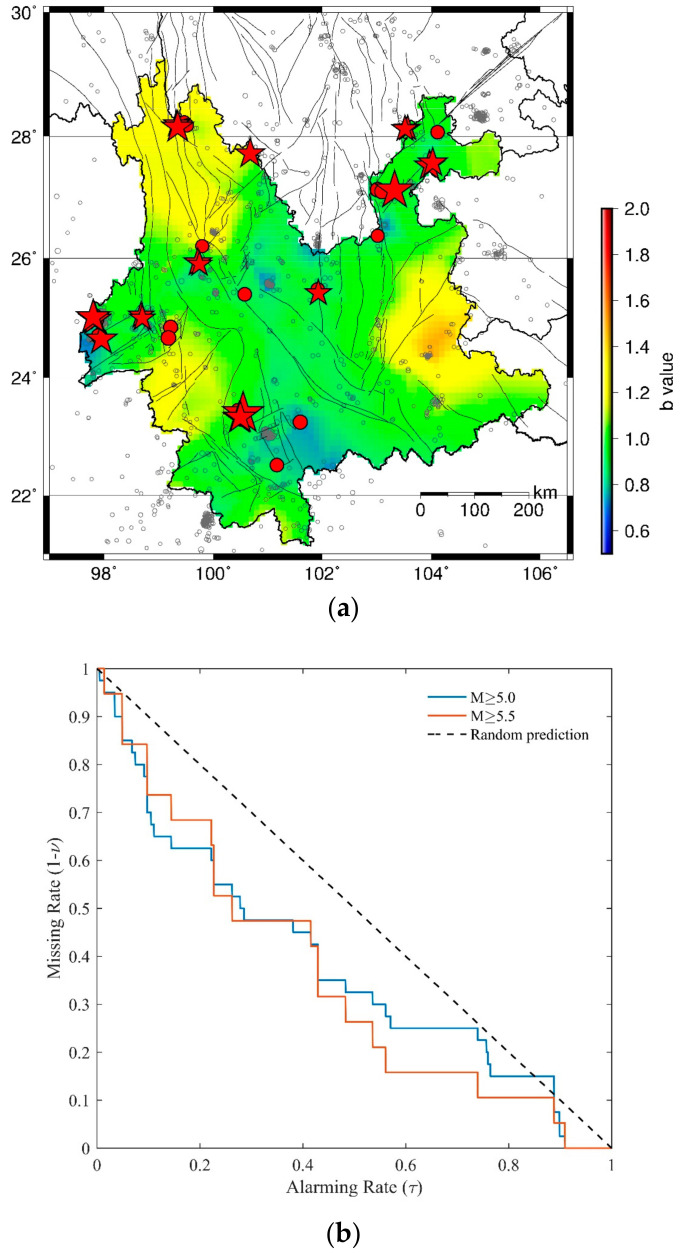

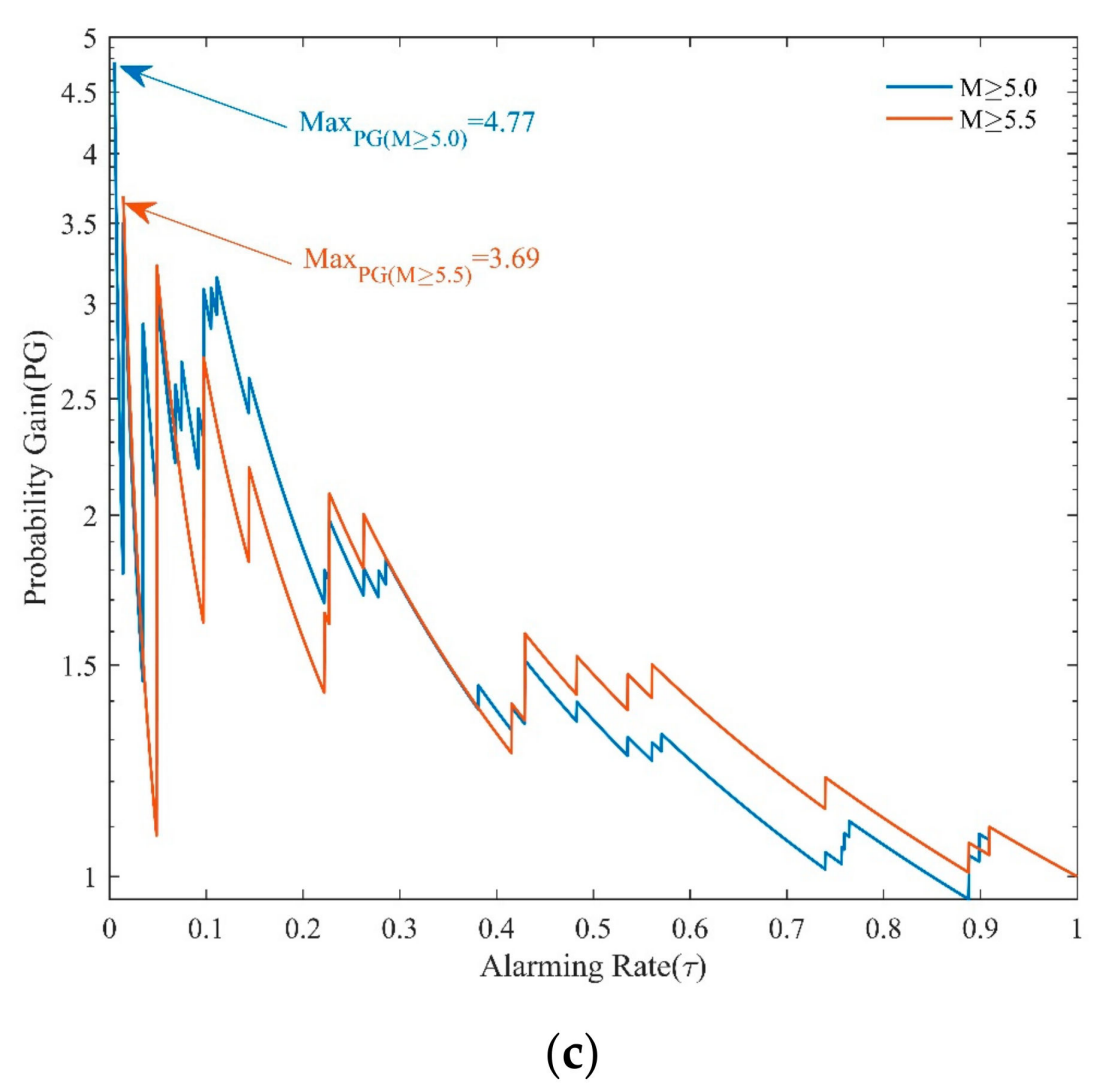
(**a**) Spatial distribution of *b* values in January 2005–December 2009. Black circles present the earthquake events with M ≥ Mc during January 2005–December 2009. Red dots show locations of events with 5.0≤M<5.5 and red stars show locations of events with M≥5.5 in Yunnan province during January 2010–December 2014. The size of the symbol is scaled to the magnitude. (**b**) MED of forecast performance using the *b* values in (**a**). The earthquake number $N_{M\geq 5.0}=40$ and $N_{M\geq 5.5}=19$. The blue line gives the result for M≥5.0 events and the red line shows the result for M≥5.5. (**c**) The PG variations of the predictions in (**b**).

**Figure 5:**
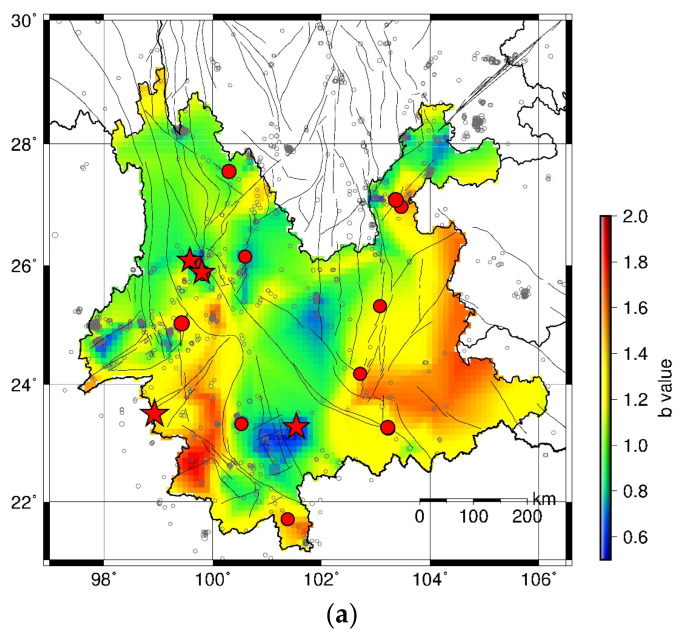

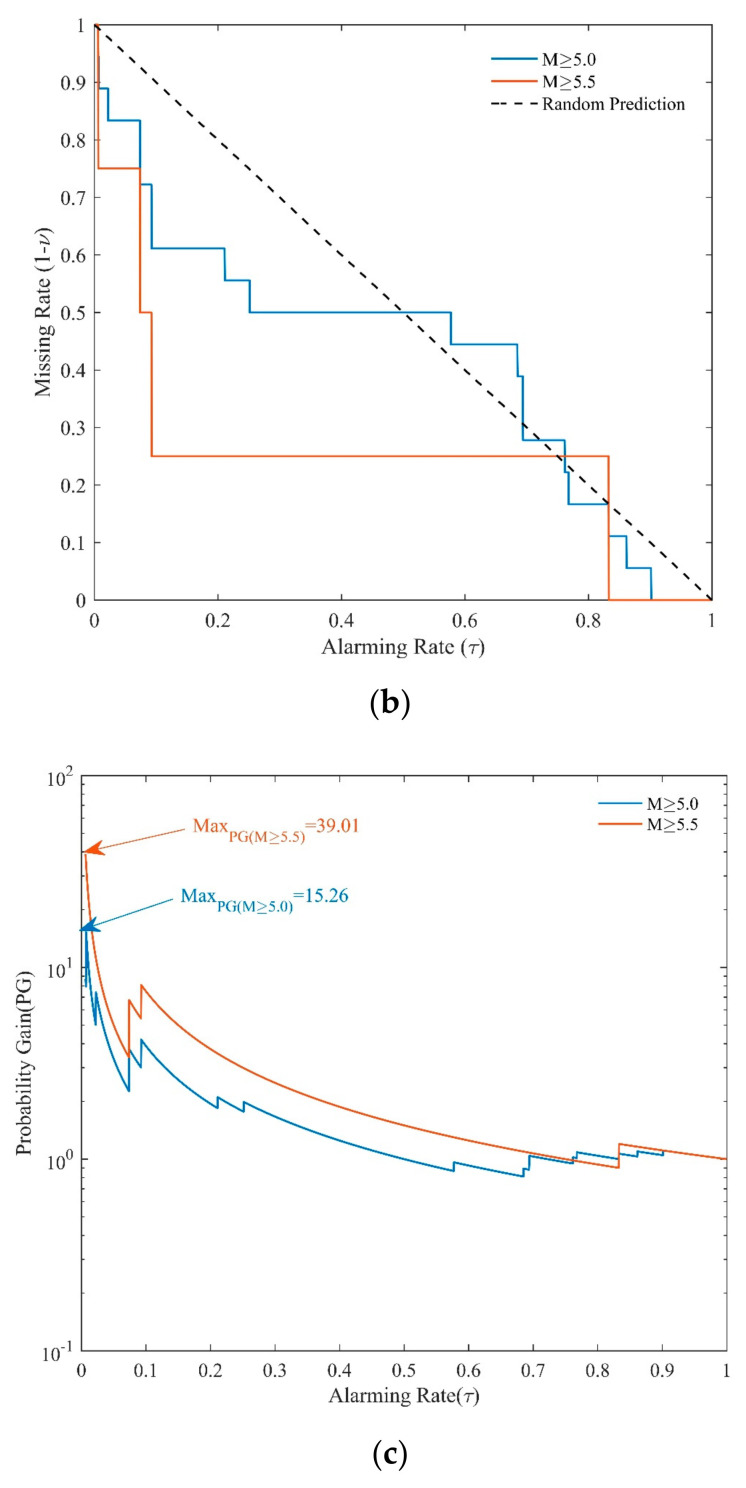
(**a**) Spatial distribution of *b* values in January 2010–December 2014. Black circles present the earthquake events with M ≥ Mc during January 2010–December 2014. Red dots show locations of events with 5.0≤M<5.5 and red stars show locations of events with M≥5.5 in Yunnan province during January 2015–December 2019. The size of the symbol is scaled to the magnitude. (**b**) MED of forecast performance using the *b* values in (**a**). The earthquake number $N_{M\geq 5.0}=18$ and $N_{M\geq 5.5}=4$. The blue line gives the result for M≥5.0 events and the red line shows the result for M≥5.5. (**c**) The PG variations of the predictions in (**b**).

**Figure 6:**
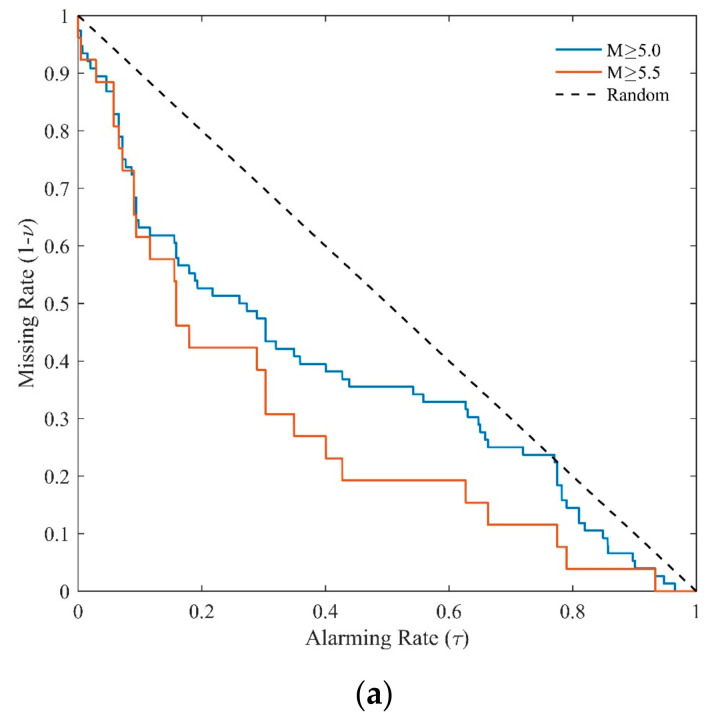

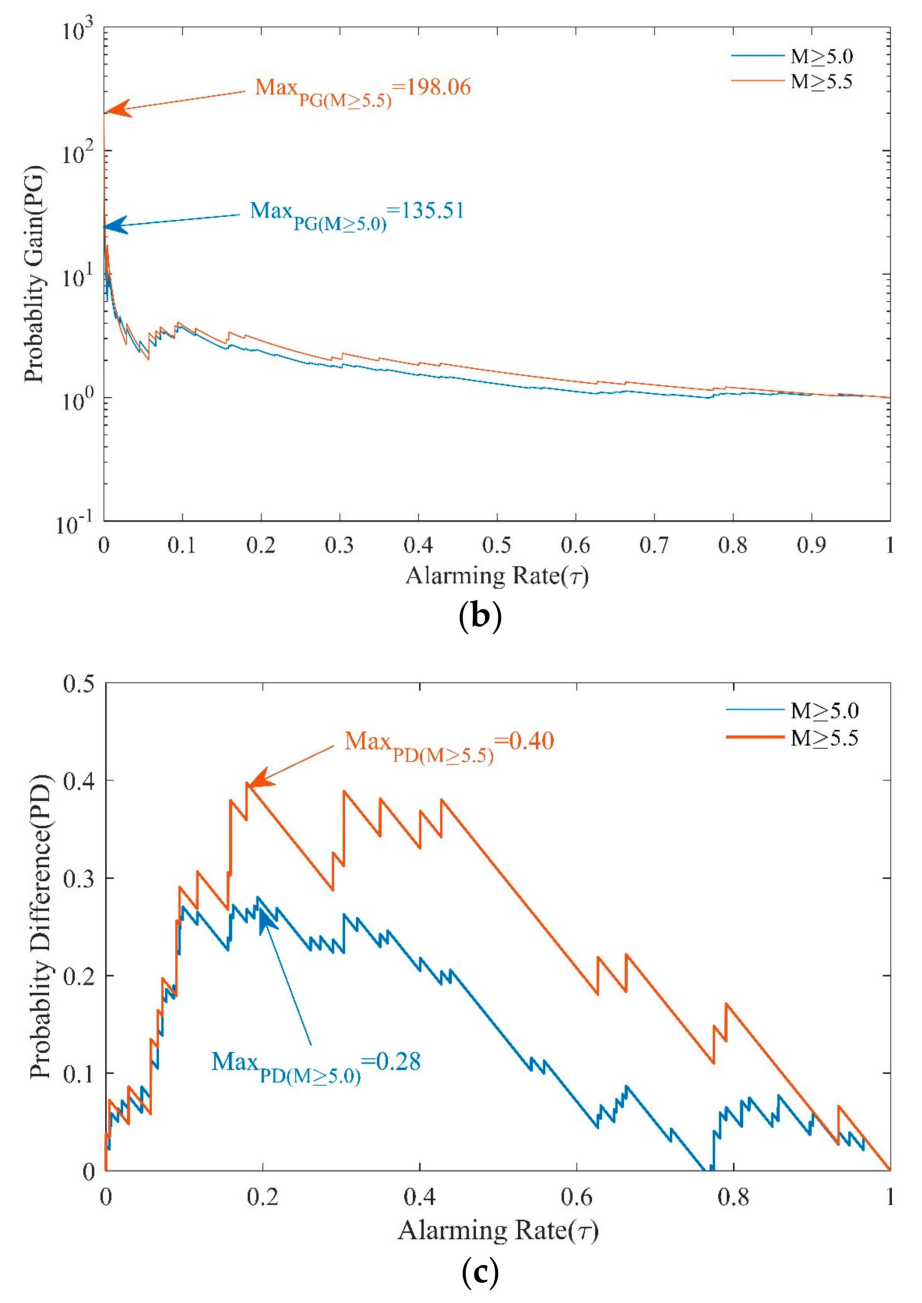
(**a**) MED of comprehensive forecast performance during January 2005–December 2019. The earthquake number $N_{M\geq 5.0}=76$ and $N_{M\geq 5.5}=26$. The blue line gives the result for M≥5.0 events and the red line shows the result for M≥5.5. (**b**) The *PG* variations of the predictions in (**a**). (**c**) The *PD* variations of the predictions in (**a**).

The HIST-PPM method is applied to earthquake catalog with M ≥ 3.0 in each 5-year period in Yunnan Province. The results of the spatial *b* values during January 2000–December 2004, January 2005–December 2009, January 2010–December 2014, and January 2015–December 2019 are shown in Figure 3a, Figure 4a, Figure 5a and Figure 6b, respectively. The moderate–large earthquakes in the subsequent period are also plotted in Figure 3a, Figure 4a and Figure 5a for comparison. Earthquakes with M ≥ 5.5 are presented with red stars, and earthquakes with 5.0 ≤ M < 5.5 are shown by red dots. It can be found that the spatial b value changes in different time periods and moderate–large earthquakes are more likely to occur in areas with low *b* values, particularly for earthquakes with M ≥ 5.5. To quantify the precursory information in the spatial *b* values, the MED is employed to test the forecast performance. 

MED is designed for estimating the ability of earthquake forecasting and presenting relationship between the rate of space tagged as alarming earthquake and the rate of earthquakes’ failure to alarm [70,71,72]. Taking Figure 3a as an example, firstly we choose a threshold of *b* value (*bthr*), and then we alarm the grids with b value<bthr. If an earthquake in the subsequent period (i.e., January 2005–December 2009) occurs in the alarmed grid, it is counted as a precited event. Otherwise, it is counted as a missed event. Define N=the number of total grids in Yunnan, $N_{1}=the\;number\;of\;alarmed\;grids$, and the alarming rate can be given as $\tau =N_{1}/N$. Define n=the number of total events, $n_{1}=the\;number\;of\;predicted\;events$, and the earthquake detecting rate can be given as $\nu =n_{1}/n$. The earthquake missing rate is $1-\nu =1-n_{1}/n$. With the threshold *bthr* increasing from the minimum to maximum value in Figure 2a, the alarming rate changes from 0 to 1 and the earthquake missing rate decreases from 1 to 0. The MED plots the missing rate versus the alarming rate, as shown in Figure 2b. The diagonal line on which the missing rate equals the alarming rate indicates a complete random guess. If the prediction curve is under the diagonal line, the missing rate is less than the alarming rate and the prediction is better than a random guess. Otherwise, if above the diagonal line, the prediction is worse than a random guess [70,71].

For the probability gain (*PG*), computing the ratio of gain (detecting rate) to cost (alarming rate) is as follows [70,73,74]:

(10) $$PG=\frac{\nu }{\tau }$$

which is utilized to further quantify the forecasting efficiency. *PG* = 1 indicates the prediction efficiency is the same as a random guess. *PG* > 1 implies that the prediction strategy is better than a random guess. The higher the *PG*, the better the prediction performance. 

Figure 3a shows the spatial *b* value during January 2000–December 2004. Figure 3b presents the MED results using the *b* values in Figure 3a to predict the earthquakes during 2005–2009. The corresponding *PG* value is given in Figure 3c. The number of earthquake events with M ≥ 5.0 and M ≥ 5.5 are 18 and 3, respectively. It is evident that the prediction curves of both M ≥ 5.0 events (blue line) and M ≥ 5.5 events (red line) are under the diagonal, suggesting that the forecast performance is better than a random guess. Almost all the *PG* values are larger than 1. The Max *PG* for M ≥ 5.0 events and M ≥ 5.5 events are 190.72 and 572.17, respectively. 

Figure 4a shows the spatial *b* value during January 2005–December 2009. Figure 4b presents the MED results using the *b* values in Figure 4a to predict the earthquakes during 2010–2014. The corresponding *PG* value is given in Figure 4c. The number of earthquake events with M ≥ 5.0 and M ≥ 5.5 are 40 and 19, respectively. All the prediction curves are under the diagonal line and the *PG* value is above 1 except one point with an alarming rate around 0.9 on the blue line. The Max *PG* for M ≥ 5.0 events and M ≥ 5.5 events are 4.77 and 3.69, respectively.

Figure 5a shows the spatial *b* value during January 2010–December 2014. Figure 4b presents the MED results using the *b* values in Figure 5a to predict the earthquakes during 2015–2019. The corresponding *PG* value is given in Figure 5c. The number of earthquake events with M ≥ 5.0 and M ≥ 5.5 are 18 and 4, respectively. The prediction curves are under the diagonal line when the alarming rate is less than 0.5. As the alarming rate rises above 0.5, the prediction becomes close to a random guess. The Max *PG* for M ≥ 5.0 events and M ≥ 5.5 events are 15.26 and 39.01, respectively. 

From the above results it can be found that low *b* values in space can be a possible indicator of forthcoming moderate–large earthquakes in Yunnan Province, China. The prediction curves based on *b* values in the MED are under the diagonal line in general and most *PG* values are above 1. These imply that the spatial *b* value contains precursory information. However, due to the number of earthquakes samples being relatively small, the results in each testing period may lack robustness. Therefore, next we perform a comprehensive analysis by integrating the three test periods.

### 3.2. Comprehensive Forecast Performance during 2005–2019

To obtain comprehensive results, a time–space alarm model is utilized [75]. In the model, the number of time–space cells is 3433 grids × 3 time periods = 10299. Same as the process in Section 3.1, firstly we choose a *bthr*, and then we alarm the cells with b value<bthr. If an earthquake in the subsequent period occurs in the alarmed cell, it is counted as an alarmed event. Otherwise, it is counted as a missed event. In the same manner, we can compute the earthquake detecting rate, missing rate, and alarming rate.

Figure 6 show the comprehensive forecast performances of *b* value during January 2005–December 2019. The number of earthquake events with M ≥ 5.0 and M ≥ 5.5 are 76 and 26, respectively. The prediction curves of both M≥5.0 and M≥5.5 are under the random prediction line in Figure 6a. The *PG* values in Figure 6b are all above 1. The Max *PG* for M ≥ 5.0 events and M ≥ 5.5 events are 135.51 and 198.06, respectively. 

In Figure 6b, although the Max *PG* is high, in practice, it is not a good choice to issue forecasts based on the corresponding *bthr* because the missing rate will be extremely high, and most earthquakes will not be predicted. In fact, as shown in the MED, a higher alarming rate will probably lead to predicting more earthquakes and can reduce the missing rate. Meanwhile, a higher alarming rate will cause more false alarms. Thus, it is important to make a trade-off between cost (false alarm) and gain (detecting rate). To find out a more applicable solution, we employ the probability difference (*PD*):

(11) PD=ν−τ

which measures the difference between the detecting rate and alarming rate [73]. For a random prediction, the *PD* is expected to be 0. *PD* > 0 which indicates that the prediction is better than random. Figure 6c shows the *PD* variations of the two prediction curves in Figure 6a. Both are clearly above 0, indicating that the comprehensive forecast performance of the *b* value during January 2005–December 2019 is obviously better than a random guess. The Max *PD* for M ≥ 5.0 events and M ≥ 5.5 events are 0.28 and 0.40, respectively.

It is noticed that in Figure 6a, the red line is mostly under the blue line, implying that at a given alarming rate, the missing rate for M ≥ 5.5 events are lower than that of M ≥ 5.0. Similar results could be found in Figure 6b,c, suggesting a possible magnitude dependence of the forecasting performance. 

## 4. Discussion

### 4.1. The Advantage of HIST-PPM Method

The conventional grid search method is widely used for *b* value estimation. It utilizes the set of seismic events close to the grid points with a fixed number or a fixed radius, which brings an inevitable overlap of the seismic events on adjacent faults. For Yunnan province, which is crisscrossed by active faults, a radius of few tens of kilometers may cover multiple tectonic units. Because different types of faults (normal, reverse, and strike-slip) may have different *b* values [16,17,25], if earthquakes on multiple faults are mixed in the computation, the *b* values would be mis-estimated. The HIST-PPM method applies the triangulation of earthquake points and estimates the *b* value by the Bayesian method. It does not require allocation of earthquake samples and can provide a higher space coverage, even if the earthquake events are relatively rare. Thus, the HIST-PPM method may have advantages in computing spatial *b* values in the study area.

### 4.2. The Influence of Mc

**Figure 7:**
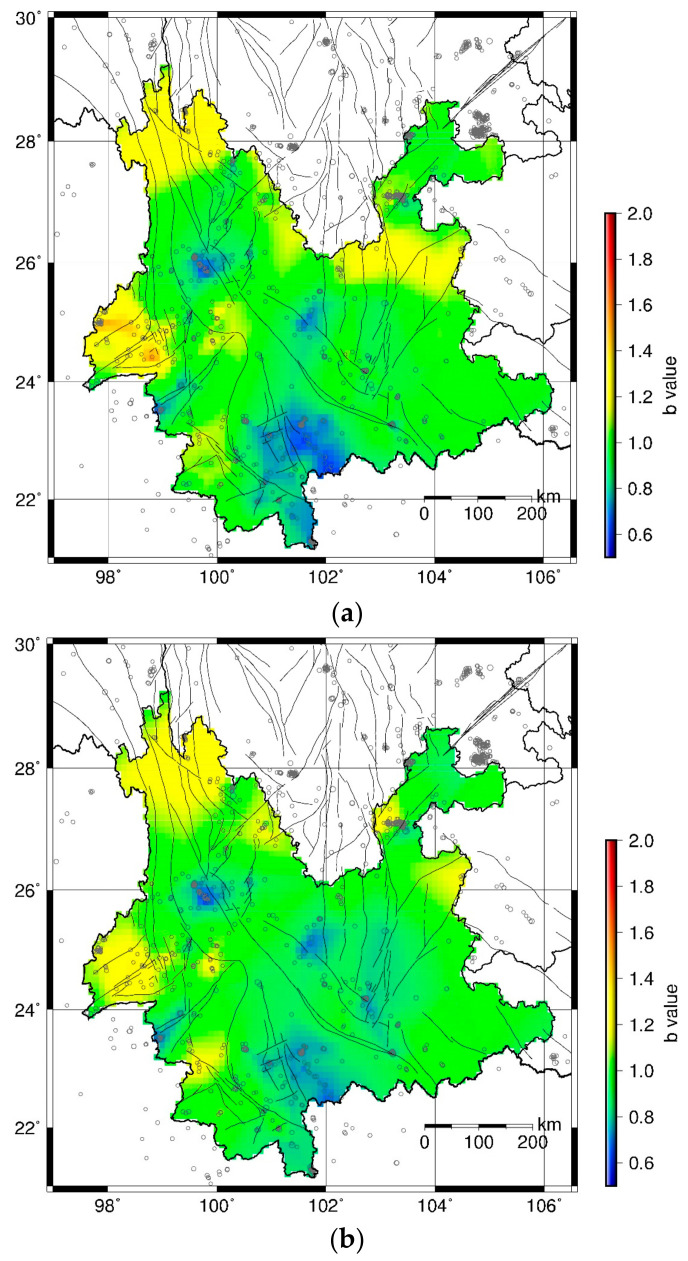

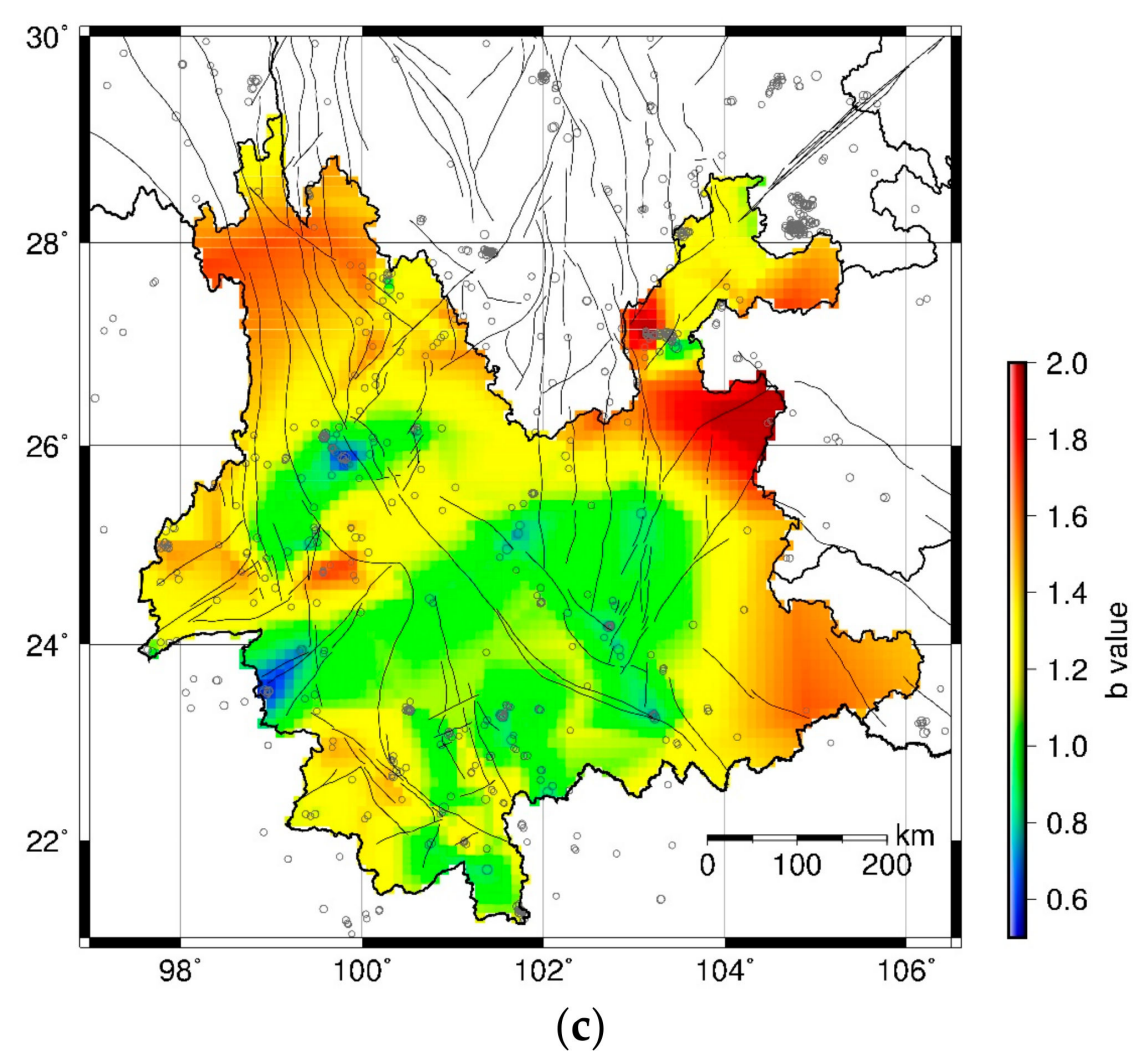
Spatial distribution of b values in January 2015–December 2019 with different *Mc*. (**a**) *Mc* = 2.8; (**b**) *Mc* = 3.0; (**c**) *Mc* = 3.2.

*Mc* is the key parameter in seismicity analysis. Different *Mc* may give different *b* values [36,60]. As shown in Figure 2b, the Max *Mc* is 2.6 in the Yunnan region during January 2000–December 2004. The seismic network is gradually improved after 2000. In general, the *Mc* decreases with time. For a fair comparison, we set *Mc* using the data in the first test period January 2000–December 2004. Considering that the MAXC might underestimate the *Mc*, we use *Mc* = 3.0 in this study. To test the influence of *Mc*, we compute the *b* values during 2015–2019, using *Mc* = 2.8, 3.0, and 3.2, respectively. The results are shown in Figure 7. It is found that the results in Figure 7a,b are quite similar. The *b* values in Figure 7c show considerable differences from those in Figure 7a,b. This may be due to the fact that the number of events with M ≥ 3.2 are small. However, the low *b* value areas show good consistency in the three figures. 

### 4.3. The Uncertainties of b Value

The HIST-PPM method applies the triangulation of earthquake locations and estimates the *b* value at epicenters by the Bayesian method. The results of the *b* value depend on the location and magnitude of earthquake events. In practice, the magnitude and location have a certain degree of error. Therefore, it is necessary to evaluate the uncertainty of the *b*-value.

According to the newly issued general ruler for earthquake magnitude (GB 17740—2017) in China, the earthquake catalog reports local magnitude (*M*_L_) if *M*_L_ < 4.5, and reports surface wave magnitude (*M*_S_) (shallow earthquake) or body wave magnitude *m*_b_ (deep-focus earthquakes) otherwise [76]. The catalog used in this study mainly contains two types of magnitudes, i.e., *M*_L_ and *M*_S_, as most earthquakes in Yunnan Province are shallow. On the other hand, the magnitude calibrating function may have errors, and the magnitude determined by different stations can be quite different. The reported magnitude is an average of the magnitudes at several seismic stations. These uncertainties of magnitude can affect the estimated *b* values. The errors of the earthquake location depend on the seismic wave velocity model, the onset time picking of seismic waves, and the number of seismic stations. Unfortunately, to the best of our knowledge, there is no systematical study available to provide an error estimation of the earthquake magnitude and location in the Yunnan region at present. 

For future study, it would be worthwhile to collect information on the uncertainties of earthquake magnitude and location so that new synthetic catalogs can be generated. Applying the HIST-PPM to the synthetic catalogs and then computing the standard deviation might be a possible way to estimate the error of the *b* value.

### 4.4. Implications and Applications

**Figure 8:**
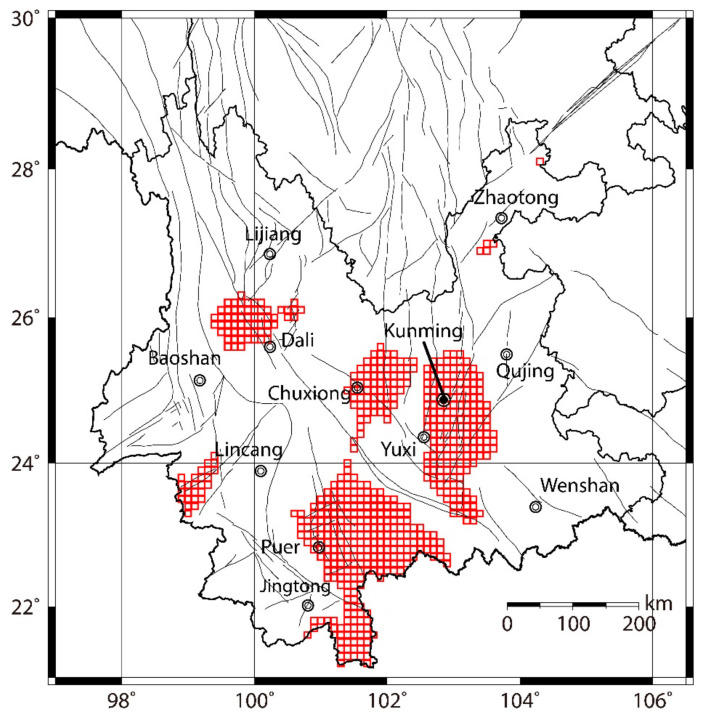
Forecast results based on the *b* values in Figure 7b with *bthr* = 0.91. The red squares indicate the alarmed areas for the period January 2020–December 2024.

As mentioned in Section 3.2, in practical application, it is important to make a trade-off between cost and gain. Therefore, we use the *PD* parameter to find out an applicable solution to the earthquake forecast in January 2020–December 2024. As our attempt, we choose the *bthr* corresponding to Max *PD* of M ≥ 5.5 as the threshold of the *b* value to issue alarms. The forecast results based on the *b* values in Figure 7b with *bthr* = 0.91 are shown in Figure 8. The red squares indicate the alarmed areas for the period January 2020–December 2024. According to the comprehensive forecast performance during January 2005–December 2019, it is expected that around 50% of M≥5.0 earthquakes and 60% of M≥5.5 earthquakes during January 2020–December 2024 will occur in the red squares. These areas include the northwest of Dali city and Chuxiong city, west of Yuxi city, and east of Puer city.

## 5. Conclusions

The HIST-PPM method is applied to the earthquake catalogs during the past two decades to reveal the spatial–temporal distributions of the *b* value in Yunnan Province, China. The spatial *b* values are calculated in each 5-year period and then are used to forecast moderate–large earthquakes (M ≥ 5.0) in the subsequent period. The forecast performance is tested by MED and the efficiency is quantified by *PG* and *PD* parameters. It is found that moderate–large earthquakes in Yunnan are more likely to occur in low *b* regions. The MED analysis suggests that there is considerable precursory information in spatial *b* values and the forecast efficiency increases with magnitude. It is concluded that the *b* value might be useful in middle- and long-term earthquake forecasts in the study area. Based on the latest five-year catalog data and the comprehensive forecast performance during 2005–2019, we provide an estimation of future earthquake locations during January 2020–December 2024.

## Data Availability

The catalog in this study is provided by the Earthquake Administration of Yunnan Province, China.
